# An Effective Intervention for Diabetic Lipohypertrophy: Results of a Randomized, Controlled, Prospective Multicenter Study in France

**DOI:** 10.1089/dia.2017.0165

**Published:** 2017-11-01

**Authors:** Catherine Campinos, Jean-Pierre Le Floch, Catherine Petit, Alfred Penfornis, Patrice Winiszewski, Lyse Bordier, Marie Lepage, Catherine Fermon, Jacques Louis, Catherine Almain, Didier Morel, Laurence Hirsch, Kenneth W. Strauss

**Affiliations:** ^1^ CH René Dubos Diabetes Service, Pontoise, France.; ^2^ Clinique de Villecresnes Diabetes Service, Villecresnes, France.; ^3^ CH Sud Francilien Diabetes Service, Corbeil-Essonnes, France.; ^4^ Université Paris Sud Diabetes Service, CH Sud Francilien, Corbeil-Essonnes, France.; ^5^ GHR Mulhouse Sud Alsace Diabetes Service, Mulhouse, France.; ^6^ H.I.A. Begin Diabetes Service, Saint Mande, France.; ^7^ C.H.B. Diabetes Service, Boulogne sur Mer Cedex, France.; ^8^ CH Victor Provo Diabetes Service, Roubaix Cedex, France.; ^9^ Hopital Belle Isle Diabetes Service, Metz, France.; ^10^ BD Diabetes Care, Rungis, France.; ^11^ BD, Le Pont de Claix, Isère, France.; ^12^ BD Diabetes Care, Franklin Lakes, New Jersey.; ^13^ BD Diabetes Care, Erembodegem, Belgium.

**Keywords:** Insulin, Injections, Needles, Subcutaneous, Lipodystrophy, Lipohypertrophy

## Abstract

***Background:*** Lipohypertrophy (LH) is highly prevalent and is potentially harmful to insulin-injecting patients.

***Methods:*** In this study, we assessed the impact of injection technique (IT) education, including use of a 4-mm pen needle on insulin-treated patients with clinically observed LH in a randomized, controlled, prospective multicenter study in France with follow-up of 6 months. Intensive education and between-visit reinforcement were given to the intervention group. Control patients received similar messages at study outset.

***Results:*** A total of 123 patients were recruited (age 52.1 ± 15.7 years; men 70.7%; body mass index >30 kg/m^2^: 34.2%; type 1: 53.7%; years with diabetes mellitus: 18.1 ± 10.5), of which 109 patients were included in the final analysis. The intervention group (*n* = 53) showed a significant decrease of total daily dose of insulin (average at baseline: 54.1 IU) at 3 months (T-3) and 6 months (T-6), reaching just over 5 IU versus baseline (*P* = 0.035). Corresponding, although not significant, decreases occurred in controls (*n* = 56); between-group differences were not significant. There were significant decreases in HbA1c (up to 0.5%) at T-3 and T-6 in both groups, with no significant differences between groups. A significant number of intervention patients improved their IT habits; about half achieved ideal IT habits by T-3 versus a quarter of control patients. By T-6, 2/3 of intervention patients achieved either ideal or acceptable IT habits, while only 1/3 of control patients did.

***Conclusions:*** Our intervention was effective in both study arms, however, to a greater degree and more rapidly in the intervention group. Widespread application of this intervention could be highly cost-effective.

## Introduction

Past studies have shown that in many patients with diabetes mellitus (DM), long-term insulin usage is associated with changes in the fatty tissue below the skin surface at injection sites, with the appearance of nodules or swollen/hardened areas.^1–7^ These lesions, called “lipohypertrophy” (LH or colloquially “lipos”), are not malignant and do not behave like tumors, but they can be unsightly and worrisome to patients. More importantly, if injections are given into LH, reduced and unpredictable absorption of insulin can result.^8^

In recent years, LH has been the subject of increasing attention and study. Numerous reports have suggested that LH is much more frequent than previously appreciated, reaching double-digit percentages of the insulin-injecting population in virtually every country surveyed.^3,9–13^ Direct causes of LH have been difficult to isolate, but there is a clear association with duration of insulin therapy (with longer times yielding higher risk), increased number of injections per day, frequent reuse of needles,^2,14–16^ and especially poor site (and within site) rotation of injections.

LH may be visible as convex distortions of the skin surface, but more commonly these lesions are found by palpating injection sites (preferably using a lubricating gel^17^). The typical findings are changes (often thickening) of the texture of the subcutaneous fat. When patients continue to inject into LH, the pharmacokinetics (PK) (absorption profile) of insulin is affected. A recent PK study using a euglycemic clamp with deliberate injections into LH showed significant blunting of insulin absorption profiles and markedly increased variability when compared to injections into adjacent normal tissue.^8^ Given these PK alterations, it is not surprising that patients with LH have, on average, higher insulin consumption rates as well as worsened glucose control, reflected in significantly higher HbA1c levels. These patients also have higher rates of unexplained hypoglycemia and glucose variability.^15,17,18^ Thus, LH increases the risk for adverse clinical outcomes and raises the cost of healthcare. Hence it is imperative that we identify effective treatments for LH.

In this study, we assessed the impact of intensive injection technique (IT) education on the 6-month evaluation of clinical parameters in insulin-treated DM patients who have clinically observed LH in a randomized, controlled, prospective multicenter study, conducted in seven diabetes centers in France. The primary objective of the study was to assess whether intensive education could lower the mean baseline total daily dose (TDD) of insulin by 2.5 IU or more in the interventional group. Other endpoints assessed the impact of this intervention on HbA1c, blood glucose values, rates of hypoglycemia and glucose variability, the size and shape of the LH lesions, the use of health service resources, overall healthcare costs, and quality of life when intervention patients were compared to controls.

## Materials and Methods

### Patients

Patients with DM1 and DM2 were recruited to the study if they had been treated with insulin for at least 1 year, were between 18 and 75 years old inclusive, and had clinically visible and/or palpable LH. Our target was to recruit 95 patients randomized to the intervention arm and 95 to the standard care arm. The study took place over four clinic visits (recruitment, T-0 [study start], T-3 [3 months ±10 days after T-0], and T-6 [6 months ±10 days after T-0]). These visits roughly corresponded to patients' usual clinical appointment intervals. A computer-generated simple randomization table assigned patients to either the intervention or standard care arms. Study personnel accessed this online table only after patients had been assessed for eligibility and had signed informed consent.

### Nurses

A study day was held for participant nurses to review the protocol, randomization schedules, and Clinical Report Forms (CRF), and to train in detection, grading, and measurement of LH. Further training was given to nurses in the intervention arm. This involved mastery of education tools specific to that arm of the study, including the use of a checklist to reinforce learnings with the patient during follow-up phone calls. Within each center, nurses were divided randomly into approximately one half who delivered only standard care and one half who delivered only intensive training. Once patients were randomized to a study arm, they were assigned to receive training and follow-up by one or the other of the cluster of nurses. The object of this clustering was to avoid contaminating bias in which, overtly or unintentionally, a nurse gave more or less training than the arm requires. Each nurse was asked not to consult across clusters as to training techniques until the end of the study.

### Intervention group

The intervention consisted of instructing patients to move injections to non-LH areas; reducing insulin doses initially by 20% to avoid hypoglycemia and then titrating to target; instructing these patients to correctly rotate within injection sites (leaving 1 cm between punctures and allowing used sites to heal for 2–4 weeks before injecting in them again); foregoing needle reuse; and switching to 4 mm needles. An array of educational tools (including brochures, grids, and a Lipobox™, which simulated the look and feel of LH lesions) was used to deliver and reinforce this training. Besides the clinic visit, this arm included frequent contact by phone and/or other electronic means after the initial training. The checklist used during these contacts included the following reminders: avoiding LH site injections, assessing condition and comfort of new injection sites, improving rotation habits and spacing injections 1 cm apart, using a new needle with every injection, adjusting insulin doses as needed, detecting the presence of hypoglycemia/glycemic variability, looking for clues that some injections may have been given intramuscular (IM), and switching to 4 mm × 32G needles to protect against IM injections. The checklist was used when reviewing care at each contact with the intensive training patients.

### Control group

Standard care meant delivering the education and follow-up, which were customary and routine at the center. This included informing patients of the presence of LH (if they were not previously aware) and stating that injections should not be given into that area. The training approach, tools, and intensive follow-up given to the intervention arm patients were not provided to the controls. In addition, at their return visit (3 and 6 months), control patients were asked if they had changed their injection habits (e.g., stopped injecting into LH, improved their rotation habits, and stopped reusing their needles) since entering the study. Those who did were analyzed separately to assess whether there were differences in outcomes compared to control patients who did not change technique, as well as to the intervention arm patients.

### Glucose measurements

Patients from both arms were required to bring their blood glucose meters to clinic at T-0, T-3, and T-6 and their nurses used Diasend™ software to download glucose values into the study database (Datavägen, Sweden; www.glooko.com/diasend). Patients were urged to perform glucose monitoring at least four times per day for the duration of the study. “Frequent unexplained hypoglycemia” was defined as hypoglycemia occurring one or more times weekly in the absence of a definable precipitating event such as a change in medication, diet, or activity. “Glycemic variability” was the presence of blood glucose oscillations from less than 3.3 mM/L (60 mg/dL) to more than 10.0 mM/L (180 mg/dL) at least thrice a week in an unpredictable and unexplained manner and evidence of such a pattern for at least the previous 6 months.

### Ethical considerations

The study was approved by the Ethics Committee according to French legislation L.1123-1 to L.1123-3 and L. 1123-6 to L.1123-13 of the French *Code de la Santé Publique* (CSP) and by the *Comité de Protection des Personnes*. The study was conducted in accordance with French law (CSP Loi 2004–806 du 9 août 2004), ICH Tripartite Directive (CPMP/ICH/135/95 adopted July 1996), EU regulations, and the original Declaration of Helsinki along with its subsequent amendments. All subjects were informed of the study purpose, requirements, and expectations in detail and all signed informed consent. Patient confidentiality was strictly ensured. Neither patients nor study personnel were paid for participating. At the end of the study, standard care patients were offered the same education, tools, and devices as those in the intervention arm.

### Statistical methods

All major parameters were investigated according to the following plan: distributions of intrasubject differences between T-0, T-3, and T-6 were compared either between intervention and standard care patients, as well as by individual arm. Values were presented as mean ± SD, median [IQR], or percent. A mixed-effect model (PROC MIXED) with repeated measures from SAS™ (Buckinghamshire, United Kingdom) was used, where subject was a random effect, with missing values accounted for using restricted maximum likelihood. A second analysis was performed where last observation carried forward model was used to replace values missing at T-3 and/or T-6 (missing values at T-0 were not replaced) and robustness of the results was investigated with a third analysis on complete patients only (patient's response variable available at T-0, T-3, and T-6). Glucose variability was analyzed in a number of ways, including coefficient of variation (CV), mean amplitude of glucose excursion (MAGE), mean of absolute glucose changes (MAG), and glucose fluctuation index (GFI).^19^ The percentages of glucose values >180 mg/dL, the percentages <60 mg/dL, and the percentages of “in range” values (60–180 mg/dL) were also calculated.

## Results

The seven centers recruited and randomized 123 subjects in total, 61 in the intervention arm and 62 in controls. Of these, 14 subjects were excluded from the final analysis: two because they were found later not to meet the inclusion/exclusion criteria (one intervention and one control), five because they did not have TDD values for T-0 (four intervention and one control), and seven because they did not have TDD values for T-3 and for T-6 (three intervention and four control). Therefore, only 109 subjects were included in the final analysis, 53 in the intervention patients and 56 in controls.

Of the 109 patients analyzed, the majority were men (72.5%); the mean age (±SD) was 52.0 ± 15.9 years; 29.4% were of normal weight (body mass index [BMI] <25 kg/m^2^), 35.8% were overweight (BMI 25–30 kg/m^2^), and 33.9% were obese (BMI ≥30 kg/m^2^); median [interquartile range] duration of DM was 17 [10; 25] years: 53.8% of the patients had type 1 DM and 87.2% had been injecting insulin for more than 3 years (Table 1). Duration of injecting insulin was similar in the two study groups. Number of injections per day was equivalent between the two arms (Table 1). All study patients had LH at the beginning of the study in at least one injection site, and ∼Ɛ in both groups were giving injections into the LH lesions at least once a day. This practice decreased in both groups, but the decrease was even more marked in the intervention patients than in controls (“A” in Table 2). The knowledge of IT was relatively high in each group at study initiation (Table 1), but a majority of patients in both groups were using needles longer than 4 mm (“B” in Table 2). The shift to the 4-mm pen needle throughout the study was more marked in the intervention arm than in controls. However, 21% of the patients in the control group shifted to the 4-mm needle during the study, although this was not expected to occur before T-6. Only about a quarter of patients in both groups were reusing needles at study start and both groups decreased this practice over time, again more in the intervention arm (“C” in Table 2). The majority of patients reported changing their IT between T0 and T6: 44 patients (83%) in the intervention group and 42 (75%) in the controls.

**Table 1.** Demographic Data at Study Entrance Randomized Patients

	*Intervention (*N* = 61)*	*Controls (*N* = 62)*	*P*
Age (year), mean ± SD	52.8 ± 15.1	51.4 ± 16.4	0.6145
Men, *N* (%)	40 (65.6)	47 (75.8)	0.2142
BMI (kg/m^2^), mean ± SD	28.4 ± 6.1	28.7 ± 6.0	0.7947
<18.5, *N* (%)	0 (0.0)	1 (1.6)	0.6838
18.5–24.9, *N* (%)	19 (31.1)	19 (30.6)	
25.0–29.9, *N* (%)	19 (31.1)	23 (37.1)	
≥30, *N* (%)	23 (37.8)	19 (30.7)	
30.0–34.9 (obesity class I), *N* (%)	14 (23.0)	9 (14.5)	
35.0–39.9 (obesity class II), *N* (%)	7 (11.5)	6 (9.7)	
≤40 (obesity class III), *N* (%)	2 (3.3)	4 (6.5)	
Age at diagnosis (year), mean ± SD	33.0 ± 16.1	34.6 ± 16.4	0.5929
Years with DM, mean ± SD	19.8 ± 11.5	16.3 ± 9.2	0.0707
Type of diabetes, *N* (%)			0.6466
Type 1	34 (55.7)	32 (51.6)	
Type 2	27 (44.3)	30 (48.4)	
Years injecting insulin (*N* = 118), mean ± SD	14.8 ± 12.8	12.5 ± 10.0	0.2652
Years injecting insulin, *N* (%)			0.0678
≤3 years	4 (6.6)	11 (17.7)	
>3 years	57 (93.4)	51 (82.3)	
Latest HbA1c at study entry (%), mean ± SD	8.3 ± 1.7	8.5 ± 1.8	0.6207
Presence of LH found by investigators, *N* (%)	61 (100.0)	62 (100.0)	1
Patients who knew they had LH?, *N* (%)	41 (71.9)	45 (73.8)	0.8222
Patient has received IT training in the past?, *N* (%)			0.1191
Yes	58 (95.1)	52 (83.9)	
No	2 (3.3)	6 (9.7)	
Don't know	1 (1.6)	4 (6.5)	
When was last IT training?, *N* (%)			0.915
Less than 6 months ago	5 (8.2)	8 (13.1)	
Between 6 and 12 months ago	9 (14.8)	9 (14.8)	
Between 1 and 5 years ago	21 (34.4)	17 (27.9%)	
Between 6 and 10 years ago	21 (34.4)	17 (27.9)	
None recorded	5 (8.2)	10 (16.4)	
Number of injections/day, *N* (%)			0.3032
1	4 (7.0)	5 (8.2)	
2	6 (10.5)	7 (11.5)	
3	2 (3.5)	7 (11.5)	
4	38 (66.7)	36 (59.0)	
5	6 (10.5)	5 (8.2)	
>5	1 (1.8)	1 (1.6)	

BMI, body mass index; DM, diabetes mellitus; IT, injection technique; LH, lipohypertrophy.

**Table 2.** Key Injection Parameters by Study Time

*Parameter*	*Study time*	*Patient answer*	*Intervention (N = 53),* n *(%)*	*Controls (N = 56),* n *(%)*
A. Still injecting into LH?	T-0	Always	8 (21.1)^a^	13 (31.7)^a^
		Sometimes	24 (63.2)	22 (53.7)
		Never	6 (15.8)	6 (14.6)
	T-3	Always	0	0
		Sometimes	6 (19.4)	11 (34.4)
		Never	25 (80.6)	21 (65.6)
	T-6	Always	0	0
		Sometimes	3 (11.1)	10 (29.4)
		Never	24 (88.9)	24 (70.6)
B. Needle length used	T-0	4 mm	13 (24.5)^b^	15 (26.8)^b^
		>4 mm	40 (75.5)	41 (73.2)
	T-3	4 mm	39 (78.0)	23 (44.2)
		>4 mm	11 (22.0)	29 (55.8)
	T-6	4 mm	39 (79.6)	27 (51.9)
		>4 mm	10 (20.4)	25 (48.1)
C. Single use of needle	T-0	Yes	38 (71.7)^c^	42 (75.0)^c^
		No	15 (28.3)	14 (25.0)
	T-3	Yes	45 (90.0)	44 (84.6)
		No	5 (10.0)	8 (15.4)
	T-6	Yes	46 (93.9)	45 (86.5)
		No	3 (6.1)	7 (13.5)

^a^ Baseline (T-0) values for the three responses (Always/Sometimes/Never) differ significantly between the two groups at *P* < 0.05; T-3 values (for these three responses) differ from those of T-0 for both groups at *P* < 0.05; T-3 values do not differ significantly from T-6 values for either group.

^b^ Baseline (T-0) values for the two needle lengths (4/>4 mm) do not differ significantly between the two groups; T-3 values (for these two lengths) differ from those of T-0 for both groups at *P* < 0.05; T-3 values do not differ significantly from T-6 values for either group; T-3 and T-6 values (for these two lengths) differ between groups at *P* < 0.05.

^c^ Baseline (T-0) values for the single use of needles (Yes/No) do not differ significantly between the two groups; T-3 values (for these two responses) differ from those of T-0 for both groups at *P* < 0.05; T-3 values do not differ significantly from T-6 values for either group.

At T-0, our control group had a mean TDD 7.1 IU higher than the intervention group. The overall average BMI did not differ significantly between intervention and controls at *T* = 0, but there were more control patients at the higher end of BMI (≥ 35) where patients tend to have higher TDD. Except for differences in mean TDD, the control patients were similar to the intervention ones at baseline on a broad range of parameters: age, gender, mean overall BMI, age at DM diagnosis, % of DM1 and % DM2, HbA1c, types of insulins used, pattern of prior IT training, frequency of LH injections, needle lengths, needle reuse, mean glucose, % BGM (blood glucose measurement) as hypoglycemia, % BGM as hyperglycemia, % BGM in range, and glucose variability (Table 1).

**FIG. 1.**
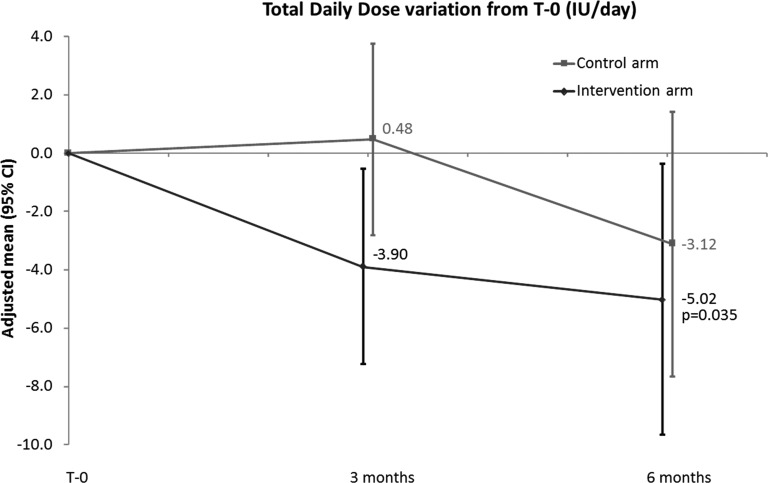
TDD by group normalized to 0 at T-0, comparisons within groups. TDD, total daily dose.

**FIG. 2.**
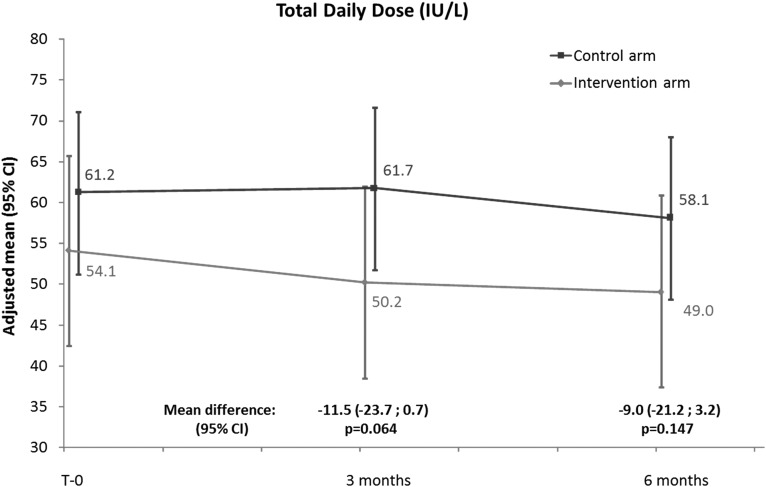
TDD by group, comparisons between groups.

The intervention group had a statistically significant decrease from T-0 of insulin TDD at both 3 and 6 months. The average decrease at T-3 was −3.90 IU/day (95% CI: 7.26, −0.54; *P* = 0.023) and at T-6 was −5.02 IU/day (95% CI: 9.68, −0.36; *P* = 0.035). In controls, the TDD tended to decrease from T-0 (0.48 IU/day [95% CI: −2.80, 3.77; *P* = 0.772] at T-3 and −3.12 IU/day [95% CI: −7.67, 1.43; *P* = 0.178] at T-6), but this was not statistically significant (Table 3 and Fig. 1). There was no statistically significant difference in change in TDD at 3 and 6 months and no difference in mean TDDs when the intervention group was compared to the control group at 3 and 6 months: −11.5 IU/day (95% CI: −23.7, 0.7; *P* = 0.064) and −9.0 IU/day (95% CI: −21.2, 3.2; *P* = 0.147), respectively (Fig. 2).

**Table 3.** Total Daily Dose Variability by Group and Time

*Parameter*	*Study time*		*Intervention (*N* = 53)*	*Controls (*N* = 56)*
TDD, by group, comparisons within groups	T-0	*N*	53	56
		Mean TDD	54.1	61.2
		95% CI	42.4, 65.7	51.2, 71.1
	T-3	Difference from T-0	−3.9	0.48
		95% CI	−7.26, −0.54	−2.80, 3.77
		*P*	0.023	0.772
	T-6	Difference from T-0	−5.02	−3.12
		95% CI	−9.68, −0.36	−7.67, 1.43
		*P*	0.035	0.178

TDD, total daily dose.

**FIG. 3.**
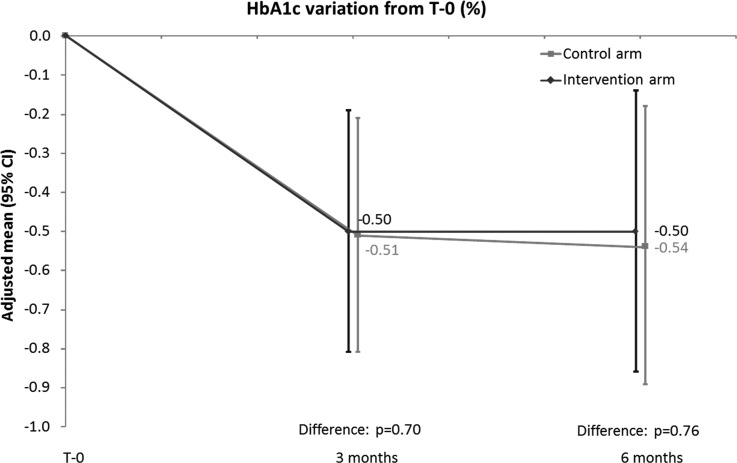
HbA1c by group and time.

Control patients started out with lower percentages of unexplained hypoglycemia (Table 4), and during the trial, the two groups moved in opposite directions: over time, controls had higher percentages, while the intervention patients had significantly lower ones. Similar patterns were found as far as glucose variability (Table 5). In both groups, HbA1c declined by ∼0.5% at 3 and at 6 months, with no significant differences between groups (Fig. 3).

**Table 4.** Hypoglycemia by Group and Time

*Parameter*	*Study time*	*Intervention (*N* = 53),* n *(%)*	*Controls (*N* = 56),* n *(%)*
Unexplained hypoglycemia by group and time	T-0	10 (25.0)	6 (13.3)
	T-3	11 (22.4)	10 (21.7)
	T-6	5 (10.9)	8 (17.8)

**Table 5.** Glucose Variability by Group and Time

*Parameter*	*Study time*	*Intervention (*N* = 53),* n *(%)*	*Controls (*N* = 56),* n *(%)*
Glucose variability by group and time	T-0	13 (32.5)	12 (26.7)
	T-3	6 (12.2)	13 (28.3)
	T-6	11 (23.9)	15 (33.3)

In terms of glucose control, there was no statistical difference (*P* = 0.461) between controls (mean BGM reading 179) and intervention patients (mean BGM reading 171) in values downloaded through Diasend from meters. In terms of the variability of BGM readings, there was no statistical difference in SD (*P* = 0.284) between controls (mean SD 74) and intervention patients (mean SD 67). In addition, no differences were seen between groups in glucose value CV, MAGE, MAG, or GFI. The percentages of glucose values >180 mg/dL, <60 mg/dL, and “in range” (60–180 mg/dL) were also not different between groups.

Despite the fact that all patients in both groups had LH at T-0 (inclusion criteria), LH could only be detected in 36 (72.0%) of the intervention patients and in 41 (78.8%) of controls by T-6. This suggests that LH lesions had regressed or disappeared in approximately a quarter of patients (statistically not significant between groups) during the course of the study.

**FIG. 4.**
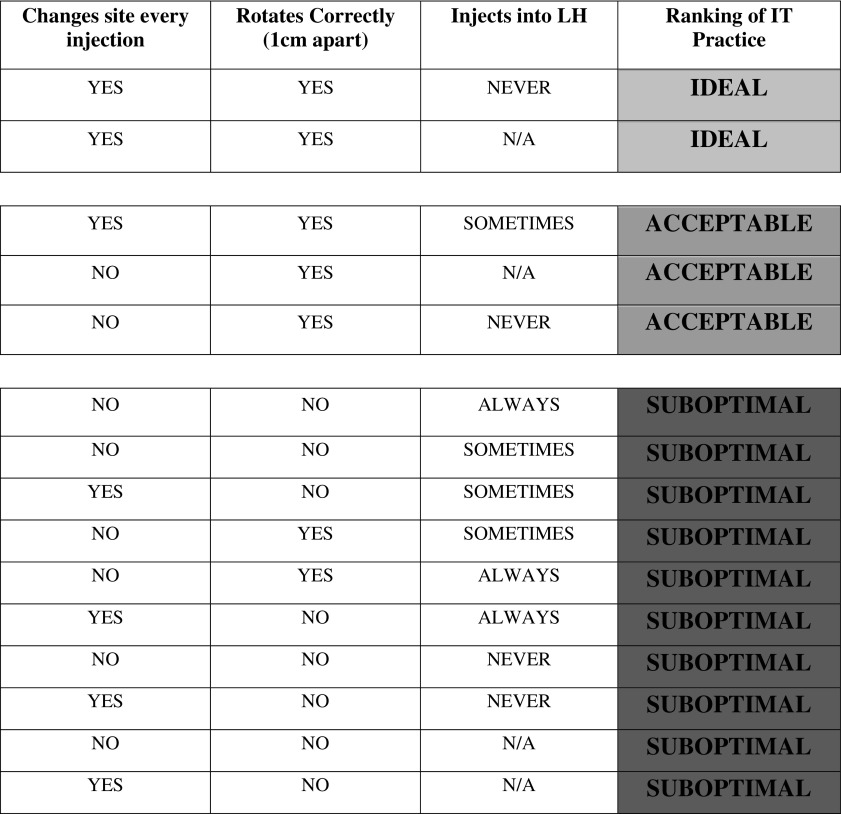
Ranking of injection technique practice by rotation and injection into LH criteria.

All subjects, regardless of arm, were assessed for behavioral changes. This included ceasing or reducing the practice of injecting into LH, improving their rotation habits by alternating sites and by spacing injections at least 1 cm apart, and reducing or stopping the reuse of needles. In each of the three study visits, patients were asked if they were currently changing their site with each injection, Yes or No. Nurses were asked to observe the rotation pattern of each patient and grade it as correct (spacing injections at least 1 cm apart) or not. Also, finally patients were asked whether they were still injecting into LH or not. Based on responses to these three questions, a ranking system on IT was devised (Fig. 4).

All subjects, without exception, scored in the “red” zone of Figure 4 at study commencement, that is, they were not alternating sites or spacing out injections, or they were injecting into LH. Three-quarters of control patients changed their IT habits during the course of the study, with 25.0% of them achieving ideal IT habits (green in Fig. 4) by T-3 and 27.1% by T-6. Compared to controls, 47.8% of intervention patients achieved ideal IT habits by the T-3 timepoint and 50.0% by T-6. Furthermore, 58.7% of intervention patients achieved either ideal or acceptable IT habits (green or yellow rankings) by T-3 and 63% by T-6, while 37.5% of control patients did so by T-3 and 35.4% by T-6.

Quality of life was assessed using the WHO-5 questionnaire. At T-0, 79% of analyzed patients reported they had an acceptable quality of life, 17% had a low quality of life, and 5% were in probable depression. No significant differences were found at 3 and 6 months compared to T-0, and no significant difference between the intervention and control groups was noted (data not shown).

## Discussion

With a disorder as prevalent as LH and as potentially harmful to patients, assessment for LH should rank high among the priorities of healthcare professionals (HCP). However, this assessment often presents technical difficulties. To look for LH, patients must disrobe and the HCP must take the time to carefully visualize and palpate all injection sites. In the usual healthcare settings, where patient visits are scheduled 15–20 min apart (and often, less), it is quite challenging to perform an appropriate assessment. Furthermore, if LH is found, targeted education and appropriate follow-up must be given to these patients. Hence, it is imperative to know which educational interventions are the most effective.

The core question of our study was as follows: when DM patients who have LH and inject into it switch injections to normal tissue sites, what are the clinical, societal, and health economic impacts? A related question is as follows: what sort of intervention can shift behaviors away from persistent LH injection and toward more optimal ITs?

The intervention group showed a statistically significant decrease of TDD of insulin at 3 and 6 months, the latter reaching just over 5 IU/day, a decrease of 9.3% from a baseline of just over 54 units. Slightly less, but still impressive, although not statistically significant, TDD decreases occurred in controls. In addition, there were significant decreases in HbA1c (of ∼0.5%) at both 3 and 6 months in both intervention and control patients, with no significant differences between the groups. We found no significant differences between the two groups for mean blood sugar, hypoglycemic or hyperglycemic episodes, or measures of quality of life. How do we interpret these nearly equivalent effects on so many key parameters between groups? Some impact can obviously be attributed to the “study effect,” but other factors are at play.

To qualify for this study, all patients had to have LH, and a large majority of patients in both arms knew they had LH at study entry (Table 1). Most were injecting into it at that time, despite having a relatively high level of knowledge of proper ITs. Nurses spent a great deal of time at each study visit inspecting, palpating, measuring, and assessing the texture of LH. This drew patient attention to these lesions and provoked questions from patients, including controls, to the HCP as to the etiology of LH, its timing, significance, consequences, and treatments. The HCP could not withhold answers to these questions, including to controls. For ethical reasons, as patient advocates and for professional ones—as this was considered standard care already in most centers in France—all patients received information at baseline about optimal ITs in regard to LH.

This, we believe, is the main reason we found overlap and sometimes equivalency in clinical outcomes. Instead of discrediting the value of the intervention, we believe these findings support and reinforce it. Control patients had similar, if less profound, falls in TDD; and these changes came later (between the T-3 and T-6 timepoints) than in the intervention group (where they mainly occurred between T-0 and T-3). Hence, the intensive education appears to hasten and enhance this effect. It is also telling that LH lesions regressed or disappeared in approximately a quarter of patients in both arms by T-6.

Another important point is that, during the study, many patients in the control group shifted to the 4-mm needle, half of them using it at T-6, compared to 80% in the intervention group. Use of the 4-mm length needle is recommended due to its proven equivalence to longer needles in terms of glycemic control, with less pain and greater preference by patients, and reduced risk of inadvertent IM injection at all injection sites (facilitating greater site rotation).^20–23^ On the one hand, this could explain, at least in part, the nonsignificant differences observed between groups. On the other, it underlines the importance of the use of such needles, which is now usual care by educators and prescribers in France for the management of LH.

At baseline, our control patients had mean TDD values 7.1 units higher than the intervention patients. However, there were considerably more control patients in the highest BMI category, ≥35 kg/m^2^. When evaluated by a large number of other parameters, controls and intervention group did not differ significantly (Table 1). Given this homogeneity between groups, we believe the increased TDD in the control group was due to a random inclusion of a number of higher BMI subjects (≥35) who may have been more insulin resistant and required higher TDD. Regardless, TDD differences between the two groups changed only slightly over the course of the study, ending at 9.0 IU, NS different at 6 months.

Significant and sustained behavioral change is one of the major challenges DM patients face. One of the cardinal questions of this study was whether our intervention could promote significant behavioral change. To assess this, we asked control patients if they had changed their injection practices during the study and three-quarters said they had. However, it was unclear what such changes entailed. Therefore, we developed a ranking scheme against which to judge this change, not only for controls but for the entire group (Fig. 4). Applying specific behavioral criteria (changing injection sites, correct rotation, and no injection in LH), it was found that all enrolled patients started off with suboptimal IT habits (every single patient scored in Darker tone).

However, during the course of the study, that behavior changed. A significant number of control patients improved their IT habits, with almost a quarter of them achieving ideal IT habits (Lighter tone in Fig. 4), most by T-3. About half of intervention patients achieved ideal IT habits, again mostly by the T-3 timepoint. About 2/3 of intervention patients achieved either ideal or acceptable IT habits (Lighter or Intermediate tone rankings) by T-6, while only a third of control patients did. All signs indicate that education was effective in both groups, however, to a lesser degree and less rapidly in the control group—similar to the TDD findings, noted above. We consider this behavioral change to be one of the most remarkable achievements of this study, hence the title of this article, “an effective intervention.” Additional follow-up will tell how sustainable this change is and what effect it has on clinical outcome parameters.

Our study does have a number of weaknesses. Foremost among them is the question of cross-pollination of control groups with the intervention (or a watered-down version thereof) and what education can be ethically withheld from such patients. It is generally agreed that ethical equipoise supports withholding information from control patients when there is reasonable doubt as to the efficacy of an intervention based on the absence of clinical trial data. That was not the case in this trial.

Older studies, although not as methodologically rigorous as this one, have shown that injections into LH lead to unstable blood glucose values^15^; LH arises in the presence of incorrect injection rotation practices and excessive needle reuse^14,16^; LH can be treated and possibly prevented by correct rotation and optimized practices such as reducing needle reuse; and correct rotation is safest (i.e., minimal IM injection risk) when the shortest pen needles (currently 4 mm) are used.^18,24^ Furthermore, a recent, very rigorous pharmacokinetics/pharmacodynamics study clearly showed reduced and highly variable insulin uptake from injections into LH, with impaired postprandial control of blood glucose.^8^

These results were generally known by the nurses in France who took part in the study and corrective procedures had become part of their standard practice. Many of the study patients also knew about LH and the possible significance. That they were not following these guidelines is supported by the large numbers who continued to inject into LH at study commencement and who were not correctly rotating their injections. That the intervention was effective is supported by the improvements in TDD, HbA1c, unexpected hypoglycemia, and glucose variability rates. That the improvements were greater and more rapid in the intervention than the control arm shows that, despite cross-contamination, the interventions we used were effective.

Another important limitation in our study was that we recruited only 2/3 of our target sample size. This suggests our study is likely underpowered and that we may be observing a type 2 statistical error (failing to detect a difference that may in fact exist). Conversely, it also suggests that significant differences we found may be even stronger than those suggested by *P*-values reported in this study. We recognize that there is a degree of speculation, but the reductions in TDD and HbA1c might have been even larger and some differences between groups could have been significant had we recruited and analyzed 190 subjects instead of 109, and if the control group had not been as IT educated as they were.

Our study only followed patients for 6 months. This raises questions of the sustainability of the positive results. Consequently, we asked the center that recruited the most patients (CORBEIL-ESSONNES) to compare the TDD and HbA1c values at close of study (T-6) with the very latest values, now 20 months after T-0. Thirty of the original 35 patients had available current follow-up data. We observed that there was *continued and significant improvement* in both parameters. The mean TDD at T-6 for the 30 evaluable patients was 61.6 IU. By T-20, it had fallen an additional 21.0 units to 40.6 IU (*P* = 0.002). The mean HbA1c at T-6 was 8.08 and it fell to 7.88 by T-20. When mean individual differences were analyzed, a similar trend was found: −19.1 IU for TDD and −0.15% for HbA1c when T-20 was compared to T-6. These trends held whether the patients had randomized to the intervention or control arm (but by T-20, control patients had already been offered the same intervention as patients in the intervention arm). Although this follow-up assessment fell outside the bounds of the study and data were only available on a subset of patients, these dramatic trends encourage the belief that our intervention is not only effective, but is sustainable.

Finally, the health economic implications of our study are quite important. Among the 800,000 insulin- injecting patients in France,^25^ ∼50% have LH according to the recent Injection Technique Questionnaire (ITQ) survey.^13,26^ The ITQ showed that the average TDD in France was 59.0 IU/patient/day. Our study shows that educational intervention for LH can reduce insulin consumption by on average 5 IU/day per patient, or ∼8.5% of the average TDD in France. Since the average cost of one IU of insulin in France is 0.034 euros, this extra spending due to LH, on the order of 25 million euros per annum, could be saved in France. These amounts are only direct and immediate savings and do not take into account additional mid- and long-term savings from improved DM management, lower resource usage, and possibly fewer complications.

There is, of course, a cost to delivering education. Each patient encounter in the intervention arm took an average of 90 min of nursing time, with the T-0 one taking up to 120 min. About half that time was spent filling out the CRF, which in our study was particularly dense. Hence, we estimate that delivering intensive education takes at least 45 min, and costs ∼30 euros in France. Each control encounter took on average 60 min, with T-0 taking up to 90. The CRF took 45 min; so standard care education takes ∼15 min, which costs 10 euros in France.

Further study on the impact of intensive education in LH is underway in a similar Chinese, randomized controlled study with a population that does not have the extensive IT knowledge that our nurses and patients had (L. Hirsch, personal communication). It is hoped that this study will continue to clarify the role of intensive injection education in the treatment of LH and its impact on key clinical outcomes.

## Conclusion

Our intervention was effective in both arms of the study, however, to a greater degree and more rapidly in the intervention group. Widespread application of this intervention could be highly cost-effective.

## Supplementary Material

Supplemental data
