# Supplementary material for: An Effective Intervention for Diabetic Lipohypertrophy: Results of a Randomized, Controlled, Prospective Multicenter Study in France
Source: Diabetes Technol Ther. 2017 Nov 1;19(11):623–32. doi: 10.1089/dia.2017.0165 (PMC5750448; doi:10.1089/dia.2017.0165)
Supplement: Supplemental data [file Supp_Data.pdf]

Supplementary Data

Electronic File of Participating Nurses and Doctors

Participating centers

Center	*Location*	*Intervention arm,* N *(% of total)*	*Control arm,* N *(% of total)*	*Total,* N *(% of total)*
1. CH Victor Provo	Roubaix	2 (3.3)	2 (3.2)	4 (3.3)
2. Hôpital Belle Isle	Metz	13 (21.3)	14 (22.6)	27 (22.0)
3. GHR Mulhouse Sud Alsace	Mulhouse	9 (14.8)	8 (12.9)	17 (13.8)
4. CH Sud Francilien	Corbeil	17 (27.9)	20 (32.3)	37 (30.1)
5. CH de Boulogne	Boulogne Sur Mer	10 (16.4)	12 (19.4)	22 (17.9)
6. CH René Dubos	Pontoise	6 (9.8)	4 (6.5)	10 (8.1)
7. HIA Begin	St. Mandé	4 (6.6)	2 (3.2)	6 (4.9)

Boulogne sur mer: Infirmières Sabine Genillier, Caroline Stevenard, Guénola St Maxent, Marinne Lamarre, Deborah Descamps, Sabrina Haussoulier, Valerie Falempin, Hélène Deprat, Virginie Holuigue; and Doctors Abdelkader Benotmane, Aagathe Poussin and Marie Lepage.

Corbeil: Infirmières Isabelle Cassier, Marie Hélène Petit, Mouna El Makni; and Doctors Pr Alfred Penfornis and Catherine Petit.

Metz: Infirmières Françoise Tonnelier, Rachel Hofschneider; and Doctor Jacques Louis.

Mulhouse: Infirmières Anne-Emmanuelle Couty, Julia Duda, Florence Walter, Frédérique Sittler and Doctors Patrice Winiszewski, Liviu Serb and Anne-Sophie Arbey.

Pontoise: Infirmières en consultation: Sophie Fautrat, Marie-Noëlle Marcellin, Christine Jullien, Lise Rousseau. Infirmières en hospitalisation: Sabrina Gendre, Paulette Anouman, Christine Groheux, Dominique Cravier; and Doctor Catherine Campinos.

Roubaix: Infirmières Peggy Degouysse and Caroline Gautier; and Doctors Catherine Fermon and Nathalie Fauvergue.

Saint Mandé: Infirmières Elise Hamon, Julia Hun, Virginie Cottereau, Yasmine Dhane, Vicky Bernard, Virginie Daniel, Florence Held; and Doctors Pr Lyse Bordier, Pr Manuel Dolz and Cyril Garcia.
